# Depression scores and quality of life of vertiginous patients, suffering from different vestibular disorders

**DOI:** 10.1007/s00405-022-07366-y

**Published:** 2022-04-18

**Authors:** András Molnár, Stefani Maihoub, Panayiota Mavrogeni, László Tamás, Ágnes Szirmai

**Affiliations:** 1 Department of Otolaryngology and Head and Neck Surgery, Semmelweis University, Szigony u. 36., 1083 Budapest, Hungary; 2 Tóth Ilona Health Service Clinical Medical Institute, Görgey Artúr tér 8., 1212 Budapest, Hungary

**Keywords:** Vertiginous patients, Depression, Quality of life, Dizziness Handicap Inventory, Beck Depression Inventory, Symptom Checklist-90-Revised

## Abstract

**Purpose:**

To contrast the quality of life (QoL) impairment and depression scores of patients suffering from different vestibular disorders.

**Methods:**

301 patients were examined due to vertiginous complaints at the Neurotology Centre of the Department of Otolaryngology and Head and Neck Surgery of Semmelweis University. These patients completed the Hungarian version of the Dizziness Handicap Inventory (DHI), the Beck Depression Inventory, and the Symptom Checklist-90-Revised questionnaires.

**Results:**

According to neurotological examination, the distribution of the different diagnoses was as follows: Menière’s disease (*n* = 101), central vestibular disorders (*n* = 67), BPPV (*n* = 47), vestibular neuritis (*n* = 39), other unilateral peripheral vestibulopathy (*n* = 18), PPPD (Persistent Postural-Perceptual Dizziness) (*n* = 16), vestibular migraine (*n* = 8), and vestibular Schwannoma (*n* = 5). The results of the DHI questionnaire have indicated worsened QoL in 86.4%, out of which 33.6% was defined as severe. The Beck scale has shown depressive symptoms in 42.3% of the cases, with severe symptoms in 6.3%. Significantly higher total DHI and Beck scale results were observed in patients with central vestibular disorders, vestibular migraine, PPPD and peripheral vestibulopathy, contrasted to the results of the other four diagnosis groups. The onset of the symptoms did not significantly affect the severity of QoL worsening and depression symptoms.

**Conclusion:**

In this study, the QoL of vertiginous patients was worse in general, with the occurrence of depression symptoms. A difference was observed in the case of the values of patients with different vestibular disorders, indicating the importance of different factors, e.g., central vestibular compensation, behavioural strategies and psychological factors.

## Introduction

Vertigo is a common complaint, with a 17–30% prevalence, according to population-based studies [1]. At a neurotological centre, benign paroxysmal positional vertigo (BPPV), Menière’s disease (MD) and vestibular neuritis (VN) can be mentioned as the most frequent diagnoses [2]. Although, other disorders, just like vestibular migraine (VM) [3], Persistent Postural-Perceptual Dizziness (PPPD) [4], and other vestibulopathies, can also influence the patients’ quality of life (QoL) and be associated with psychiatric comorbidities. Psychiatric comorbidities are present in around 30–50% of the patients suffering from dizziness or vertigo [5]. Among them, one of the most common ones were defined as depression [6], with a prevalence of 4–62%, based on the literature [7]. Due to vertigo or dizziness, impaired QoL often occurs. Psychiatric comorbidities may further worsen the vertiginous patients’ QoL. Moreover, due to the QoL impairment, reduced daily activities, and inability to work, patients can present psychological distress [8]. Previous studies have observed differences in the QoL impairment and psychiatric comorbidities of patients suffering from different vestibular disorders [9, 10]. Therefore, besides analysing patients’ QoL and depression scores, the present study aimed to analyse the QoL and depression scales in different disorders resulting in vertigo or dizziness.

## Methods

### Participants

In this prospective investigation, 301 patients were enrolled. The basic demographical data of the subjects, along with the results of the Dizziness Handicap Inventory (DHI) and Beck depression (BDI) questionnaires, are summarised in Table 1. These patients underwent a complete neurotological examination and have filled out the following questionnaires. The distribution of the different diagnoses, according to neurotological examination, was as follows: MD (*n* = 101), central vestibular disorders (*n* = 67), BPPV (*n* = 47), VN (*n* = 39), other unilateral peripheral vestibulopathy (*n* = 18), PPPD (*n* = 16), VM (*n* = 8), and vestibular Schwannoma (*n* = 5). Several diagnoses can be found in the background of other unilateral peripheral vestibulopathies, including, e.g., ototoxicity or complication of acute or chronic suppurative otitis media, labyrinthitis, trauma, perilymph fistula, etc. As these aetiologies occurred in a low number of cases, they were grouped into one category. The central vestibular disorders group included several diagnostic entities as follows: cerebral ischaemia (27%), vertebrobasilar insufficiency (23%), cerebellar infarction (19%), posterior circulation stroke (17%), other central causes (7%), multiple sclerosis (5%) and tumours of the brainstem and fourth ventricle (2%).

**Table 1:** Basic demographical data, BDI and DHI results

Age (mean ± SD years)	56.1 ± 13.4
Gender (male/female)	82/219
Duration of the symptoms (vertigo or dizziness; mean ± SD months)	93.2 ± 76
DHI, *n* (mean ± SD)	
Normal handicap (0–14)	41 (7.5 ± 4.1)
Mild handicap (16–34)	90 (23.3 ± 5.2)
Moderate handicap (36–52)	69 (42.9 ± 4.5)
Severe handicap (54 <)	101 (71.5 ± 9.3)
Functional score (mean ± SD)	17.43 ± 9.5
Physical score (mean ± SD)	10.5 ± 5.1
Emotional score (mean ± SD)	13.74 ± 8.4
BDI, *n* (mean ± SD)	
Normal (0–5)	172 (2.3 ± 1.3)
Mild (6–11)	85 (7.8 ± 1.5)
Moderately severe (12–15)	25 (12.3 ± 2)
Severe (15 <)	19 (21 ± 2)

The study was approved by Semmelweis University Regional and Institutional Committee of Science and Research Ethics: SE RKEB – 203/2021.

### Dizziness handicap inventory

The DHI questionnaire is a commonly used self-administered questionnaire for assessing QoL impairment resulting from vertigo and dizziness, validated for different languages. The questionnaire contains 25 questions, with possible answers of ‘yes’ (4 points), ‘sometimes’ (2 points) and ‘no’ (0 points). The questions are divided into physical (11 questions), functional (16 questions), and emotional (10 questions) groups. The scoring of the results is done in the following way: 0–14 points indicate a normal handicap, 16–34 mild, 36–52 moderate, while a score over 54 points severe QoL worsening. All subjects have filled out a Hungarian version of the DHI [11, 12].

### Beck depression inventory

To analyse the severity of the depressive symptoms, the shortened, 13-item version of BDI was applied, which was previously reported as a reliable questionnaire for a Hungarian sample [13]. The patients can give answers from ‘0’ (‘not at all’) to ‘3’ (‘very much’), with a maximal score of 39. 0–5 means no depressive symptoms, scores of 6–11 indicate mild, 12–15 indicate moderately severe, and 16 and above indicate severe depression [14].

### Symptom checklist-90-revised

The Symptom Checklist-90-Revised (SCL-90-R) questionnaire can examine a broad range of symptoms and psychopathologies, including besides questions regarding depressive symptoms, e.g., somatisation, hostility, phobic anxiety, paranoid ideations, etc. The questionnaire included 90 questions; for each, the patient can give answers from ‘0’ (‘not at all’) to ‘4’ (‘extremely’). The subjects who were enrolled in this investigation have completed a Hungarian version of the questionnaire, and the scores of the depressive symptoms (10 questions) were analysed [15].

### Statistical analysis

IBM SPSS Statistics V24 software was used for data processing. Shapiro–Wilk test has indicated that our data was not normally distributed; therefore, the Mann–Whitney *U* test was used to detect a statistically significant difference. The significance level was set up as *p* < 0.05. Boxplot diagrams were included to illustrate the results. Simple linear correlation and Spearman correlation tests were applied to analyse correlation . A *p* value under 0.01 has indicated a significant correlation.

## Results

Table 1 shows the basic demographical data of the patients, along with the summarisation of the DHI and BDI results.

As shown in Table 1, the investigated patients were predominantly females, mostly in their fifties. The total DHI score indicated impaired QoL in 86.4% of the patients, with severe QoL worsening in 33.6%. The highest mean values were detected in the functional DHI subscore, followed by emotional scores. According to the results of BDI, depression was present in 42.3%, of which was in the severe range in around 6.3% of the patients.

Dot curves were drawn, and correlation tests were applied to analyse whether the duration of the different disorders, i.e., the time from the onset of the symptoms (i.e., vertigo or dizziness) up to the completion of the questionnaires, influences the scores or not.

**Fig. 1:**
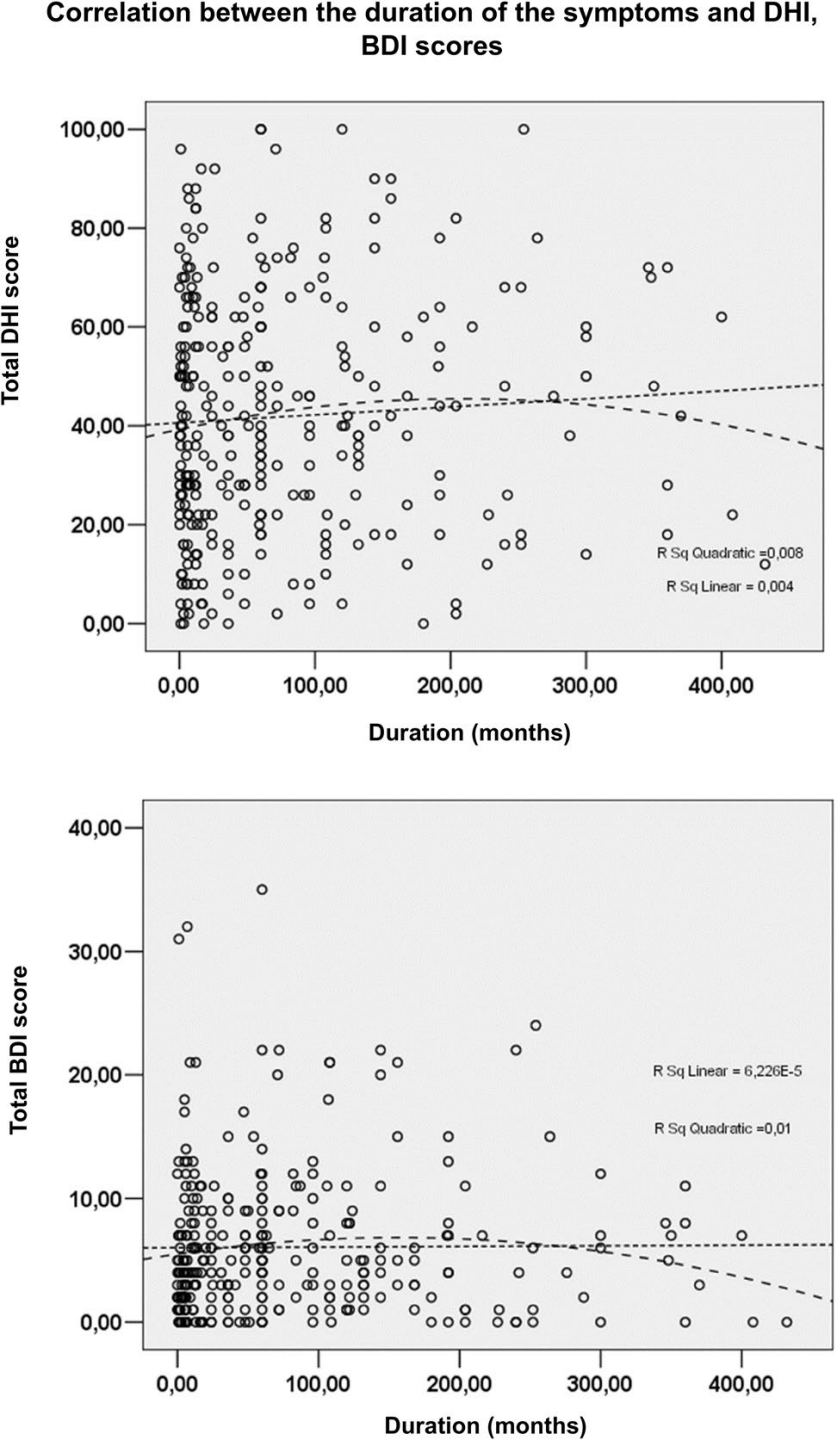
Correlation between the time since the onset of the symptoms (i.e., vertigo or dizziness) and the scores of the questionnaires

As shown in Fig. 1, no linear correlation was observed between the duration of the disorders and the total BDI and DHI scores, either. The Spearman test indicated correlation neither in the case of DHI (rho = 0.05, *p* = 0.468), nor the case of BDI (rho = 0.030, *p* = 0.658) scores; thus, a non-linear correlation was not observed either. Therefore, it can be concluded that the duration of the symptoms has no significant influence on QoL and the occurrence of depressive symptoms.

**Fig. 2:**
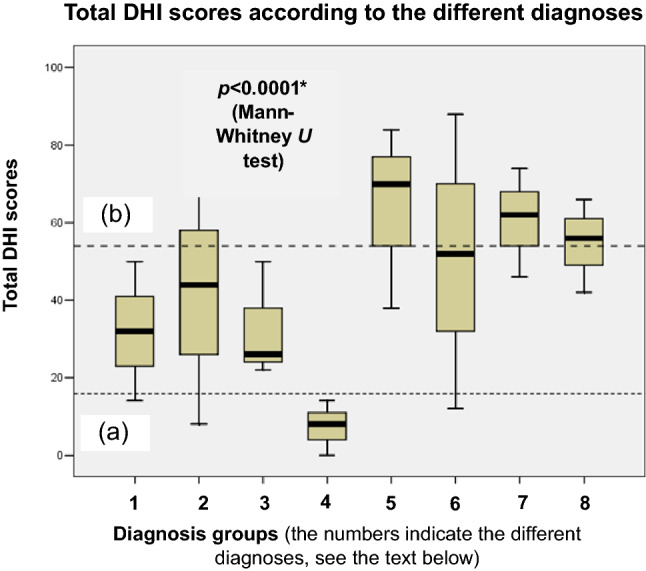
Boxplot showing the distribution of the total DHI values. Black lines in the boxes: median values, box: the middle 50% of the data, whiskers: upper and lower 25%. **a** Normal range (0–14 points), **b** severe range (54+) of the total DHI scores. 1: BPPV, 2: MD, 3: VN, 4: vestibular Schwannoma, 5: central vestibular disorders, 6: VM, 7: PPPD, 8: other unilateral peripheral vestibulopathy. The Mann-Whitney U test was used to detect differences between the examined parameters (*p<0.05)

The distribution of the total DHI scores of the patients suffering from the different disorders is shown in Fig. 2.

As shown in Fig. 2, lower values of total DHI scores were detected in the case of patients suffering from BPPV, MD, VN, and vestibular Schwannoma (Group 1). In the case of Schwannoma patients, all DHI scores were in the normal range, indicating no QoL worsening. In terms of central vestibular disorders, VM, PPPD and other unilateral peripheral vestibulopathy (Group 2), higher total DHI values were observed, and about 50% of the scores were found in the severe range. When the statistical analysis was carried out using the Mann–Whitney *U* test, a significant difference was detected between Groups 1 and 2 (*z*-score: − 4.23, *p* < 0.0001*), indicating significantly higher values in Group 2 (i.e., central vestibular disorders, VM, PPPD and other unilateral peripheral vestibulopathy). Therefore, patients suffering from the disorders as mentioned above have reported significantly worse QoL.

**Fig. 3:**
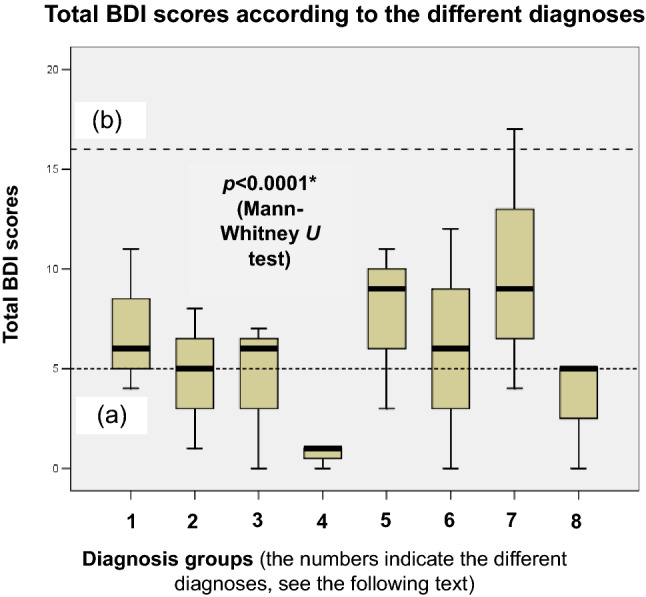
Boxplot showing the distribution of the total BDI values. Black lines in the boxes: median values, box: the middle 50% of the data, whiskers: upper and lower 25%. **a** Normal range (0–5 points), **b** severe range (16+) of the BDI scores. 1: BPPV, 2: MD, 3: VN, 4: vestibular Schwannoma, 5: central vestibular disorders, 6: VM, 7: PPPD, 8: other unilateral peripheral vestibulopathy. The Mann-Whitney *U* test was used to detect differences between the examined parameters (*p<0.05)

Figure 3 shows the same analysis for the total BDI scores.

As shown in Fig. 3, for the total BDI scores, roughly the same distribution was detected as in the case of the total DHI scores. Higher BDI values were observed in Group 2, indicating higher BDI scores of patients with central vestibular disorders, VM and PPPD. Only patients suffering from other unilateral vestibulopathies presented lower BDI values than for total DHI. According to statistical analysis, which was conducted using the Mann–Whitney *U* test, the difference between the two groups was statistically significant in this case as well (*z-*score: − 4.33, *p* < 0.0001*). This result indicates that besides the higher DHI values of this group the severity of depressive symptoms was more expressed as well.

**Fig. 4:**
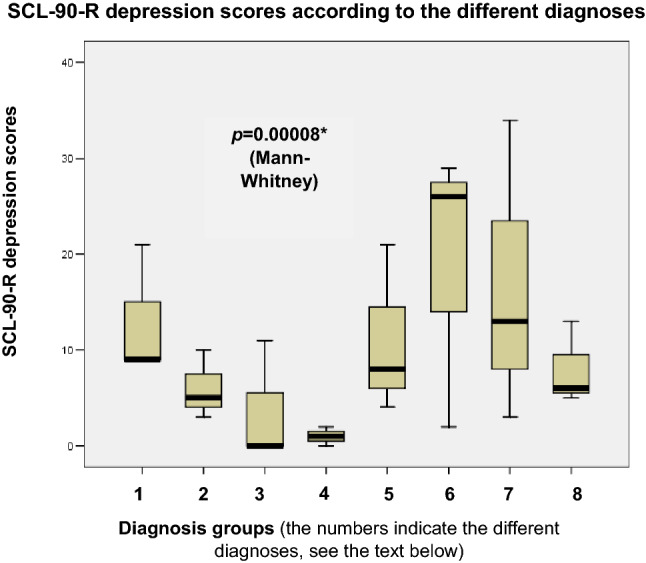
Boxplot showing the distribution of the depression scores according to the SCL-90-R questionnaire. Black lines in the boxes: median values, box: the middle 50% of the data, whiskers: upper and lower 25%. 1: BPPV, 2: MD, 3: VN, 4: vestibular Schwannoma, 5: central vestibular disorders, 6: VM, 7: PPPD, 8: other unilateral peripheral vestibulopathy. The Mann-Whitney U test was used to detect differences between the examined parameters (*p<0.05)

The same analysis was conducted for the depression scores according to the SCL-90-R questionnaire. The results are shown in Fig. 4.

Figure 4 shows that higher depression scores based on the SCL-90-R questionnaire were observed in terms of central vestibular disorders, VM, PPPD and other unilateral peripheral vestibulopathies. When the values were contrasted using the Mann–Whitney *U* test, a statistically significant difference was detected (*z*-score: − 3.93, *p* = 0.00008*). This result also indicates similar scores and depression severity, based on BDI and SCL-90-R, respectively.

## Discussion

In the present study, many  subjects suffering from vertigo were involved. The different diagnoses made it possible to analyse the differences between the several groups. This is of great importance, as a wide range of vertigo disorders is presented at a neurotologic centre. In this study, the patients were not examined in the acute phase of vertigo but rather on an appointment basis; therefore, the distribution of the different diagnoses was quite different than in other investigations [2], suggesting higher rates of MD and central vestibular disorders and BPPV was only third in the line. According to the results of the DHI questionnaires, 86.4% of the patients have reported impaired QoL, and significantly higher DHI values were detected in patients suffering from central vestibular disorders, VM, PPPD, and other unilateral peripheral vestibulopathies, compared to the results of those suffering from the other disorders. BDI has indicated depression symptoms in 42.3% of the patients, with nearly the same distribution as observed with DHI regarding the diagnoses. Previous investigations have also contrasted the values mentioned above in different disorders. For instance, Goto et al. concluded that depressive disorders were more prevalent in patients with sudden deafness and migraine-associated dizziness [9]. According to another investigation, patients suffering from central vestibular disorders have reported the highest DHI values, and patients with primer functional dizziness presented significantly higher DHI values than those with peripheral vestibular disorders [16]. In the present study, the BDI values of those suffering from PPPD and central vestibular disorders were also significantly higher, than the values of the other diagnosis groups. Zhu et al. have concluded that the emotional, physical, and functional aspects of vertigo had more significant effects on patients with VM than those with BPPV. According to their analysis, DHI scores significantly correlated with depression and anxiety scales [10]. In the present investigation, VM patients also reported significantly higher DHI and BDI values contrasted to other vestibulopathies, including BPPV, which is in concordance with the aforementioned study results. The results of Möhwald et al. indicated significantly higher DHI values in patients with acute peripheral vestibular disorders than with central ones [17]. This contradicts our results, which have shown significantly lower DHI and BDI values for most peripheral vestibular disorders than central ones. Although, in their study, patients with acute vestibular symptoms examined at the Emergency Department were enrolled, while most of our patients were not in the acute phase of the disorders but were investigated on an appointment basis at a tertiary referral centre. However, our analyses found no correlation between the onset of the symptoms, and therefore the duration of the disorders and DHI and BDI values. MD was defined as the most frequent diagnosis and is known as a chronic, progressive disorder presenting with episodic vertigo. A meta-analysis regarding MD found that about 50% of the patients suffer from depression [18]. QoL worsening in MD patients caused by the disorder was also reported [19, 20]. Moreover, a study concluded that vertigo complaints were the most intrusive symptoms, surpassing other MD symptoms, just like tinnitus or hearing loss. Additionally, vertigo was most strongly associated with depression and DHI scores [19]. Previous studies showed an increased prevalence of affective disorders in episodic vertigo disorders compared to vestibulopathies with non-episodic vertigo complaints [21]. In the present investigation, patients with episodic (i.e., MD, BPPV, and VM) and acute or chronic persistent (i.e., central vestibular disorders, PPPD, VN, and unilateral peripheral vestibulopathies) symptoms were also enrolled; however, higher DHI and BDI values were found in the non-episodic vertigo syndromes group. This is in contrast with previous findings and highlights the complexity of the background of the QoL impairment and psychiatric symptoms. According to previous findings, VN also significantly affects the patients’ QoL; a moderate impact was reported [22].

The differences in the DHI and BDI values amongst the different disorders cannot be explained only by the vestibular deficit caused by them. As previously reported, there is no correlation between the severity of the vestibular hypofunction, based on objective vestibular testing and QoL and depressive or anxiety symptoms. A study has concluded that the symptom severity and DHI values were not correlated with the results of clinical vestibular tests but corresponded with several psychological factors (i.e., depression, anxiety, cognitive and behavioural responses etc.) [23]. Another study observed no correlation between the DHI scores and high- and low-frequency vestibulo-ocular reflex deficits, respectively. Patients suffering from central vestibular disorders were found to report the highest DHI values, and functional dizziness also showed higher DHI values, contrasted to those suffering from peripheral vestibular disorders. In conclusion, it was stated that more than 96.5% of the DHI score variances resulted from unaccounted factors, just like central compensation or neurocognitive behavioural factors [16]. After vestibular deficits, central compensation is a highly complex and vital process [24]. Central vestibular compensation involves several anatomical structures of the central nervous system (i.e., vestibular nuclei, vestibulocerebellum, midbrain, dorsolateral/anterior thalamic nuclei, and posterior inferior vestibular cortex) [25], and is influenced by other factors, including behavioural strategies, socio-cultural background, age, physical activity, etc. Vertigo or dizziness influences everyday tasks and can result in psychosocial consequences. Moreover, somatisation, i.e., the increased attention to the symptoms, can also be responsible for a worsened handicap [26]. Individual coping strategies have also been suspected to be responsible for differences between vestibular tests and the handicap of the patients [27]. The factors mentioned above can influence the central compensation and, therefore, the QoL and occurrence of psychiatric comorbidities, independently from the objective results of the vestibular tests. Hence, although QoL questionnaires and psychiatric symptoms are not specific or sensitive to any vestibular disorders, and therefore, they cannot replace vestibular testing, they still can provide important information regarding the functional and psychological difficulties of vertiginous patients.

A limitation of the present study can be that the result of the previous psychiatric/psychological examinations was not considered. Therefore, it was not investigated whether the patient has two simultaneous disorders. Although, since all vertigo patients have filled the questionnaires, this population was examined in detail.

## Conclusion

The results of the present research observed significantly impaired QoL in patients with different vertigo disorders, along with the co-occurrence of psychiatric symptoms, presenting themselves as depression. The DHI and BDI scores varied amongst the different diagnosis groups, indicating a complex background of the presented symptoms after vestibular damage. Therefore, besides the objective vestibular testing, the self-administered questionnaires may also have an important role.
